# Warm temperatures during cold season can negatively affect adult survival in an alpine bird

**DOI:** 10.1002/ece3.5715

**Published:** 2019-10-25

**Authors:** Jules Chiffard, Anne Delestrade, Nigel Gilles Yoccoz, Anne Loison, Aurélien Besnard

**Affiliations:** ^1^ Ecole Pratique des Hautes Etudes (EPHE) Centre d'Ecologie Fonctionnelle et Evolutive (CEFE) UMR 5175 Centre National de la Recherche Scientifique (CNRS) PSL Research University Montpellier France; ^2^ Centre de Recherches sur les Ecosystèmes d'Altitude (CREA) Observatoire du Mont Blanc Chamonix France; ^3^ Laboratoire d'Ecologie Alpine (LECA) CNRS Université Grenoble Alpes Université Savoie Mont Blanc Grenoble France; ^4^ Department of Arctic and Marine Biology UiT The Arctic University of Norway Tromsø Norway

**Keywords:** carry‐over effect, climate change, cold‐adapted species, corvid, demography, mountain, *Pyrrhocorax graculus*, seasonality, sex‐specific survival, vertebrate

## Abstract

Climate seasonality is a predominant constraint on the lifecycles of species in alpine and polar biomes. Assessing the response of these species to climate change thus requires taking into account seasonal constraints on populations. However, interactions between seasonality, weather fluctuations, and population parameters remain poorly explored as they require long‐term studies with high sampling frequency. This study investigated the influence of environmental covariates on the demography of a corvid species, the alpine chough *Pyrrhocorax graculus*, in the highly seasonal environment of the Mont Blanc region. In two steps, we estimated: (1) the seasonal survival of categories of individuals based on their age, sex, etc., (2) the effect of environmental covariates on seasonal survival. We hypothesized that the cold season—and more specifically, the end of the cold season (spring)—would be a critical period for individuals, and we expected that weather and individual covariates would influence survival variation during critical periods. We found that while spring was a critical season for adult female survival, it was not for males. This is likely because females are dominated by males at feeding sites during snowy seasons (winter and spring), and additionally must invest energy in egg production. When conditions were not favorable, which seemed to happen when the cold season was warmer than usual, females probably reached their physiological limits. Surprisingly, adult survival was higher at the beginning of the cold season than in summer, which may result from adaptation to harsh weather in alpine and polar vertebrates. This hypothesis could be confirmed by testing it with larger sets of populations. This first seasonal analysis of individual survival over the full life cycle in a sedentary alpine bird shows that including seasonality in demographic investigations is crucial to better understand the potential impacts of climate change on cold ecosystems.

## Graphical abstract

We tested whether seasonality influence survival in a long‐lived alpine bird, before covariate analysis in a multi‐event framework. We highlight a critical survival period for adult females during the transition between spring (the end of cold season) and summer. Low female survival was associated to warm winter and spring temperatures, we discuss the sensitivity of the alpine ecosystem to spring condition from a new point of view and the importance of accounting for seasonality in these highly seasonal environments.

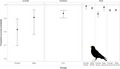

## 1 INTRODUCTION

High latitudes and high elevations are the regions on Earth most exposed to current and future climate change (Nogués‐Bravo, Araujo, Errea, & Martinez‐Rica, 2007; Pepin et al., 2015; Yoccoz, Delestrade, & Loison, 2010). In these cold ecosystems, climate seasonality is a predominant constraint on species' lifecycles (Körner, 2003; Korslund & Steen, 2006; Martin & Wiebe, 2004). In this context, predicting species' responses to future climate change relies heavily on their sensitivity to seasonal variations in a range of ecological constraints. However, interactions between seasonality, weather fluctuations and population parameters such as age‐ and sex‐specific survival remain poorly understood (Hallett et al., 2004).

Animals living in cold regions have a short breeding season, which is usually synchronized with the brief flush of resources. While many polar and alpine species escape harsh environmental periods by migrating or hibernating, others migrate only short distances, or even stay in the same range throughout the year. To survive winter periods, these cold‐adapted species often rely on metabolic, morphological, or behavioral adaptations, such as accumulated fat reserves, body insulation, or trophic flexibility (Hart, 1962; Martin, 2001). In all cases, reproduction and survival are likely to be affected differently by the variation in environmental conditions in each season. For example, warmer springs and summers have been shown to positively influence reproduction in common alpine birds (Meller, Piha, Vähätalo, & Lehikoinen, 2018; Saracco, Desante, Siegel, Helton, & Stock, 2019), but to negatively impact the survival of common birds in a wider temperate context (Pearce‐Higgins, Eglington, Martay, & Chamberlain, 2015). The impact of some environmental conditions can also be delayed to “critical periods” in the seasonal cycle through carry‐over effects. For example, spring conditions, which largely determine available forage resources for large herbivores (Bischof et al., 2012; Pettorelli, Pelletier, Von Hardenberg, Festa‐Bianchet, & Côté, 2007), have a direct effect on juvenile survival, and carry‐over effects on the survival of capital breeders the following winter, as winter survival largely relies on fat accumulation during the previous spring and summer (Harrison, Blount, Inger, Norris, & Bearhop, 2011; Loison, Jullien, & Menaut, 1999; Loison & Langvatn, 1998). In long‐lived species, high and stable adult survival is counterbalanced by high variability in younger age classes, thus survival parameters of each age class should be investigated, as well as the potential drivers of this variability (Gaillard & Yoccoz, 2003).

Predicting the impact of climate change on long‐lived terrestrial vertebrates living in highly seasonal environments is difficult due to the combination of immediate and delayed consequences of seasonality on demographic traits. Estimating such parameters in marked free‐living populations over the long‐term is the most reliable way to identify critical survival periods for individuals and the determinants of temporal variation on survival. Grosbois et al. (2008) pointed out that most demographic studies with a hypothesis based on seasonality have tested the correlation between seasonal weather covariates and annual survival estimates (e.g., Sillett & Holmes, 2002). While such studies focusing on annual survival clearly indicate that weather covariates during certain periods of the year are more influential than others, they do not allow the identification of when animals are actually at risk of mortality. This may be achieved by estimating season‐specific demographic parameters and assessing how they co‐vary in line with environmental variations (Grosbois et al., 2008).

In long‐lived terrestrial vertebrates, an additional complexity is that individuals may vary in their ability to survive depending on characteristics such as age (Gaillard, Festa‐Bianchet, Yoccoz, Loison, & Toïgo, 2000), sex (Jorgenson, Festa‐Bianchet, Gaillard, & Wishart, 1997) and body condition (Gardner, Amano, Sutherland, Clayton, & Peters, 2016). The hierarchical status of individuals in a group has also been shown to be related to survival in many species that winter in cold ecosystems, notably because this can determine access to food, and thus body condition (Ekman, 1990; Henderson & Hart, 1995; Rézouki et al., 2016). While intrinsic factors are known to influence survival variation between individuals, whether and how these individual characteristics interact with at‐risk periods has seldom been investigated in sedentary birds (although see below for migrating birds), as long‐term studies with relatively high sampling frequency are required in order to identify seasonal patterns and investigate hypotheses regarding the environmental constraints affecting them.

In birds, seasonal survival studies have mostly been carried out on migratory species (Hostetler, Sillett, & Marra, 2015; Rushing, Ryder, & Marra, 2016), which are subject to constraints that are very different to those of sedentary species (e.g., Drent, Both, Green, Madsen, & Piersma, 2003). Seasonal survival has been studied in some sedentary alpine bird species using radio telemetry (Angelstam, 1984), which seems to suggest high winter survival. However, these studies were short‐term and based on a low number of individuals, precluding useful generalizations (Chamberlain et al., 2012). Seasonal survival patterns are still unknown for most species of cold‐adapted vertebrates (Hallett et al., 2004).

To investigate the effect of seasonality on a sedentary bird living in a highly seasonal environment, we estimated the mean effect of seasons on survival in a nonmigratory population of the alpine chough (*Pyrrhocorax graculus*) living in one of the harshest mountain environments in Europe, the massif of Mont Blanc. The study was based on 27 years of capture–mark–resighting/recapture data. The alpine chough is a long‐lived, gregarious species living in labile flocks composed of stable pairs (Holyoak, 1972). From its morphology and behavior, it is inferred that the alpine chough relies on an income breeding strategy to obtain energy for survival and reproduction (Stephens, Boyd, Mcnamara, & Houston, 2009). A flock has a strong hierarchical social structure, with adults dominating immature individuals, and males dominating females on clumped food resources (Delestrade, 1993b). Given these characteristics, and the general pattern of survival observed in long‐lived species, we expected: (a) the end of the winter season (the spring in alpine environments) to be the most critical period of the year in terms of survival for all individuals (due to low food income, high requirements and lower levels of fat reserves); (b) differences in survival between adults and younger individuals to be greater during the harsh or “critical” seasons (winter and especially spring in this case); (c) dominated adults (females and small individuals) to have lower survival than dominating adults during the harsh/critical seasons; (d) winter flock size to negatively influence cold season survival, as intraspecific competition for food is strongly correlated with the relative size of a flock, despite the numerous advantages of living in a group (Clutton‐Brock et al., 1999; Lehtonen & Jaatinen, 2016); and (e) seasonal survival rates to be influenced by weather conditions such as temperature (warm summers, cold winters, or springs) and precipitation, through direct or carry‐over effects, especially during critical survival periods.

## 2 MATERIAL AND METHODS

### 2.1 Biological model and study site

The alpine chough is a long‐lived corvid with an average lifespan in the wild of 8–10 years (maximum longevity is 19 years, see Figure S5 in Appendix S1). The species is distributed in Palearctic mountainous regions between the latitudes of 30° and 50°. Alpine choughs breed at high elevations (the nest is usually hidden in cliff crevices 800–3,800 m above sea level: Delestrade & Stoyanov, 1995a; Laroulandie, 2004) and forage over a wide range of elevations in all seasons (400–4,807 m elevation in the Alps), as food resources at high elevations can be artificially maintained by human activity (Laiolo, Rolando, & Carisio, 2001). Pairs are highly stable over years, and paired individuals can be observed together even in nonbreeding seasons (Holyoak, 1972). Males are larger than females (Delestrade, 1993a; Laiolo & Rolando, 2001, and Figure S3 in Appendix S1). These birds forage in flocks whose size can vary from 2 to 1,000 individuals, with seasonal variation (Delestrade, 1994). In summer, the median flock size in the Alps was found to be 67 individuals in one study (Delestrade, 1994). In cold seasons, individuals from different neighboring breeding areas merge together, forming flocks whose median size is about 30% larger than in summer (Delestrade, 1994). Adults and first‐year individuals are distinguished by the color of their legs (red and black, respectively). In this study, we defined three age classes: first‐year juveniles (from fledgling to first autumn), immature adults (from first winter to second autumn), and adults. Sexual maturity seems to be reached during the third year (Holyoak, 1972).

The site of the study was the northern French Alps, between 2,400 and 2,700 m above sea level (Figure S1 in Appendix S1). The seasons were defined in quarters, with “summer” from June to August, “autumn” from September to November, “winter” from December to February, and “spring” from March to May (Figure S1 in Appendix S1). In the summer season, we studied individuals at three sites (site 1: “Lac Blanc”, site 2: “Albert 1er”, site 3: “Couvercle”). Capture locations were located close to common foraging areas, picnic areas, or mountain huts. The distance between each of these sites was relatively similar (9–11 km). In winter and spring, we studied individuals in a wintering site (“Le Tour” site). This is a village with a ski resort 1,400 m above sea level and 4, 7, and 10 km away from Albert 1er (site 2), Lac Blanc (site 1), and Couvercle (site 3), respectively. Birds from the three monitored summer sites and from other unmonitored summer sites gather together at the “Le Tour” site after the first snowfalls, usually in December. They then usually stay until the last weeks of May, depending on spring phenology (Delestrade, 1994, timing of breeding, for example, fledging, varies from July 30 to August 28; this variation is important with regard to the short‐time interval favorable for breeding and is driven by spring phenology in the alpine chough).

### 2.2 Data collection

In each season, individuals were baited with apples and dried grapes, and then captured with cannon nets or clap nets (Delestrade & Stoyanov, 1995a). Captured individuals were ringed with an individual combination of three or four colored plastic bands. During these capture sessions, marked individuals were resighted from up to 50 m using a telescope. No captures or resightings were performed during the autumn season in any year, nor in the spring of 2010. The first ringing season was the winter of 1988, and the last studied season was the summer of 2014, resulting in 80 “season–year” capture occasions. Capture and resighting efforts varied between sites and seasons, ranging from 1 to 37 days (mean = 16 days, Figure S2 in Appendix S1). Sex was determined by observation (when a male fed its female mate), by genetic analyses (from blood samples) or using a discriminant function analysis performed on biometric variables (wing, tail and tarsus length, position of nostril, and bill width, all measured during capture), which correctly classified 93% of adults (Delestrade, 2001).

### 2.3 Groups and covariates

We used tarsus length as a proxy of an individual's size, a common practice in bird studies (see Gosler, Greenwood, Baker, & Davidson, 1998 for field determination of body size in birds; Bókony, Seress, Nagy, Lendvai, & Liker, 2012 for an example on tarsus length). While tarsus length is a continuous covariate, we discretized it and implemented it as different size classes in the models, as implementing individual continuous variables for one thousand individuals in E‐SURGE (see below) would require months of calculation to obtain model convergence. We calculated the median tarsus length for each sex and classified individuals in four sex/size groups: small females, large females, small males, and large males. Each year, we calculated the flock size based on all the flock counts carried out during capture occasions at the Le Tour site (the cold season site). During the cold seasons across the study period (1988–2014), the total number of counts was 526, with a median number of counts per year of 20 (between 8 and 54). Flock size was calculated as the mean of the maximum value for each month. The quarterly mean of daily precipitation and of daily temperature were calculated from homogenized (discontinuity‐corrected) climate data provided by the Swiss National Basic Climatologic Network for the Grand Saint Bernard station (2,470 m above sea level) (see Begert, Schlegel, & Kirchhofer, 2005 for homogenization method). The quarterly mean of snowpack was calculated for each year of the study based on ISBA‐Crocus models (Vionnet et al., 2012) (elevational range 1,200–1,500 m) for winter and spring only. Lastly, the “snow anomaly” was calculated as the residual of a linear regression between snow cover and precipitation in cold seasons, so that the negative and positive values represent proxies of rainy and snowy seasons, respectively. All covariates were standardized before analysis: They are shown in Figure S3 in Appendix S1.

### 2.4 Capture–recapture data analysis

We performed a multistate goodness‐of‐fit test using U‐care (Choquet, Lebreton, Gimenez, Reboulet, & Pradel, 2009) on a simple dataset including data only on adults and specific seasons (winter and summer). These tests revealed very high over‐dispersion and trap‐dependency (Appendix S1), thus we chose to develop a more complex model in line with the structure of the dataset and the biology of the studied species. The dataset was made up of 3,115 winter captures or resightings and 3,323 spring resightings at the Le Tour site, and 2,722 resightings in summer sites, for a total of 9,160 captures/resightings. This dataset allowed us to fit a model with a relatively high number of parameters. A multi‐event capture–recapture (CR) modeling framework (Pradel, 2005) accounting for dispersal between monitored and unmonitored sites was used to test predictions (see below). In a multi‐event framework, “events” are the field observations related to the latent states of the individuals. These observations can involve uncertainty regarding the latent state, which is modeled through the observation process. For example, the choughs were monitored in three summer sites, but individuals breeding/living in unmonitored summer sites could be ringed and resighted during winter and spring. To deal with these individuals, a fourth summer site, a “ghost” site, was implemented in the model (see Tavecchia, Tenan, et al., 2016 for a similar approach). We defined individual states as a combination of age class and the site occupied in summer (Lebreton, Hines, Pradel, Nichols, & Spendelow, 2003; Lebreton & Pradel, 2002). To account for resighting heterogeneity in summer sites—due to distance to chough nests, and possible trap dependency from baiting (see Section 3)—we also defined two additional “hidden” states for individuals: those with high resighting probability versus those with low resighting probability (or “mixture” models, see e.g., Cayuela et al., 2014). The combination of age (three age classes), site (three monitored and one unmonitored site) and resighting heterogeneity classes (high and low resighting probability) resulted in 24 “living” states and one “dead” state (Figure S4 in Appendix S1).

The CR data were analyzed on a seasonal basis, that is, in quarters corresponding to the four seasons defined above. From its initial state at first capture, an individual could transit from state to state between quarter *t* and *t* + 1 according to four steps (Figure S4 in Appendix S1). The first step was the probability of surviving or not from *t* to *t* + 1. Survival could depend on an individual's state, notably its age and the summer site it occupied, and could also vary between quarters or between years. The second step was the probability of changing age class, which was not estimated and was forced to occur during the first (juvenile to immature) and second (immature to adult) autumn–winter transition. The third and fourth steps corresponded to dispersal probability, which was split into two steps (Grosbois & Tavecchia, 2003) and occurred during the spring–summer transition (Delestrade & Stoyanov, 1995b). The third step modeled an individual's departure probability (i.e., of leaving a summer site given it is still alive), and the fourth step its arrival probability (i.e., of reaching a specific summer site given that it had dispersed at the previous step). The first dispersal event occurred at an individual's immature stage. Immature departure probability from the ghost site was not estimated, as these individuals were not ringed as juveniles (by definition, there were no captures on the ghost summer site). Finally, the events matrix linked the 24 “living” states and the observations (birds were not observed dead). In summer, individuals were observed on summer sites, so the age and the site were known. Individuals using the ghost site were considered unobservable at this period. In winter and spring, all individuals, including those from the ghost site, were observed, but only at the winter site, so the summer site that an individual occupied was unknown. The models were fitted in the E‐SURGE program, which allowed model complexity to be addressed in a straightforward manner while keeping convergence time reasonable (a few days; Choquet, Rouan, & Pradel, 2009).

### 2.5 Model selection procedure step 1: seasonality

We first fit a general model in which quarterly survival probability depended on the effects of season, age class and sex and their interactions: Departure probability depended on the effects of age class and sex and their interaction, and arrival probability depended on the occupied site before dispersal and arrival in interaction with sex for each age class. Adult resighting probability was fitted using a log function of the number of days of fieldwork with recaptures accomplished during the corresponding season (recapture effort), with an intercept that differed between site, sex, and resighting heterogeneity class (for summer). The summer resighting probability on the ghost site was forced to 0. Immature resighting probability depended on the season and sex group in interaction, but did not depend on field effort, as the number of resightings was very low in this age class during cold seasons. From this first model, we tested simpler models by removing the effects of, respectively, resighting heterogeneity, and differences between sites, sex groups, and seasons for each parameter. We first simplified resighting probability, then dispersal, and finally survival. We used Akaïke information criteria adjusted for small sample sizes (AICc) to select the best models after each simplification step.

### 2.6 Model selection procedure step 2: weather covariates

After this selection procedure, we used the best model to investigate the ability of the mean sex/size group covariate, winter flock size and climate covariates (mean temperature, mean snow depth, snow anomaly, direct, or with carry‐over effects) to explain temporal variations in survival probability. Based on the results of the previous modeling step, which showed variation in each season (at least for females), but higher winter survival (see Section 3), we focused on the effect of covariates on spring and summer survival to avoid a multiplication of statistical tests since this can lead to an inflated risk of Type I errors (Rice, 1989). The total number of models with covariates was 12. Direct effects of covariates and carry‐over effects accounting for conditions during previous cold seasons were tested, hypothesizing an important effect of the overall cold season (winter and spring) on survival the following spring and summer. To test for carry‐over effects, covariate values in winter and spring were added together to create a new covariate. As the number of individuals was low in the juvenile and immature classes, we did not test the covariates on juveniles; for immature individuals, we grouped the estimates of each quarterly survivals of the same year together in a single parameter (mean quarterly survival per year), and then tested the effect of covariates on this parameter. The statistical support for a temporal covariate effect and the magnitude of this effect was assessed relative to the fit of constant and year‐dependent models using ANODEV (Grosbois et al., 2008). Thus, we preferred fixed effects to random effects for models including years as the first was needed for the ANODEV test; additionally, E‐SURGE does not allow fitting yearly random effects. We fixed the risk of erroneously accepting the effect of a covariate that did not have an effect at 10% (*α* ≤ 0.1). Finally, we ran a model in which survival was constant over quarters but depended on years in order to estimate the inter‐annual variation in survival. All parameter estimates were given with their associated 95% confidence intervals. This choice was made because of the relatively low number of years available to identify effects (only 30 time replicates for each seasonal survival estimate). We were more interested in the size and percentage of the effect of temporal variation on chough survival explained by a covariate with ANODEV than in statistical significance per se.

## 3 RESULTS

Over the 27‐year study period, 1,095 alpine choughs (226 juveniles, 110 immature individuals, and 759 adults) were banded. Individual history data were collected in 3,115 winter and 3,323 spring resightings at the Le Tour site, and 2,722 resightings in summer sites.

### 3.1 Model selection

The model selection procedure is shown in Table S2 in Appendix S1, and the best models are shown in Table 1. In terms of survival probability, the best model included the effects of sex and season in interaction on adult survival, no effects on immature survival, and a sex effect on juvenile survival. Departure probability was sex‐dependent in immature birds, but not in adults. Arrival probability depended on departure site in immature and adult birds, with an interaction with sex in adults. Resighting probability of adults in summer sites varied depending on field effort, with a different positive slope for each site, and the intercept varied with site and heterogeneity class in interaction with sex (Figure S8 in Appendix S1). Resighting probability of adults in winter and spring depended on field effort and season in interaction, but did not differ between sexes (Figure S9 in Appendix S1). Resighting probability of immature individuals only differed between winter/spring and the summer season.

**Table 1:** Best models for alpine chough survival

Model ID	Survival	N.P.	Deviance	ΔAICc	*p* ANODEV	*R* ^2^ Dev	Effect [CI 95%]
1	Spring (F): Fco (Temperature Winter + Spring)	95	22,306.69	−2.96	.025	0.28	−0.34 [−0.02 to −0.66]
2	Spring (F): Fco (Flock size)	95	22,308.42	−1.24	.093	0.16	−0.15 [0.03 to −0.39]
3	Spring (F): Fco (Snow anomaly)	95	22,309.53	−0.13	.18	–	0.23 [−0.09 to 0.56]
4	Spring (F): Ftime (YEAR)	111	22,293.19	16.2	–	–	–
5	Spring (F): Tarsus length	96	22,315.34	−0.26	–	–	0.02 [−0.03 to 0.07]
6	Spring (F): Fcst	94	22,311.69	0	–	–	–
7	J(SEX) + IM + AD(SEX*SEASON)	94	22,317.63	0	–	–	–
8	J(SEX) + IM(SEASON) + AD(SEX*SEASON)	96	22,313.78	0.22	–	–	–
9	J(SEX) + IM(SEX) + AD(SEX*SEASON)	95	22,317.43	1.83	–	–	–

Note

Models below the gray line vary in terms of sex or season effects on survival. Models above the gray line investigate drivers of spring survival in adult females. N.P. = number of estimated parameters; Deviance = model residual deviance; AICc = Akaïke information criterion corrected for small sample size; *p* ANODEV = *p* value for the ANODEV test statistic following an *F* distribution; *R* ^2^ Dev = the proportion of the variation in survival explained by the covariate. Effect: estimated slope of the correlation between survival and the covariate.

Abbreviations: AD, adults; J, juveniles; IM, immature individuals.

### 3.2 Survival

**Figure 1:**
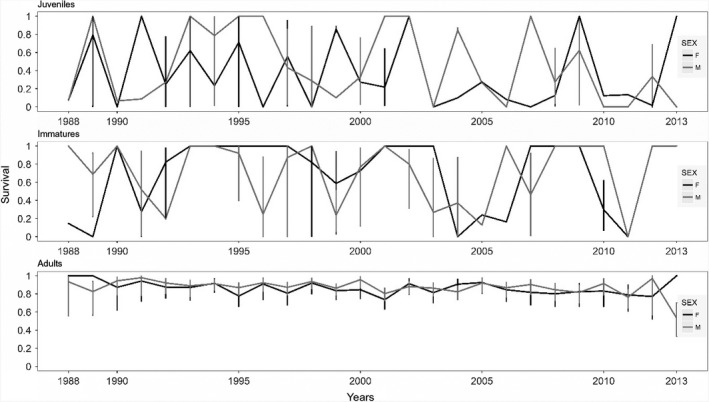
Annual survival probability of the alpine chough by age class over the study period

**Figure 2:**
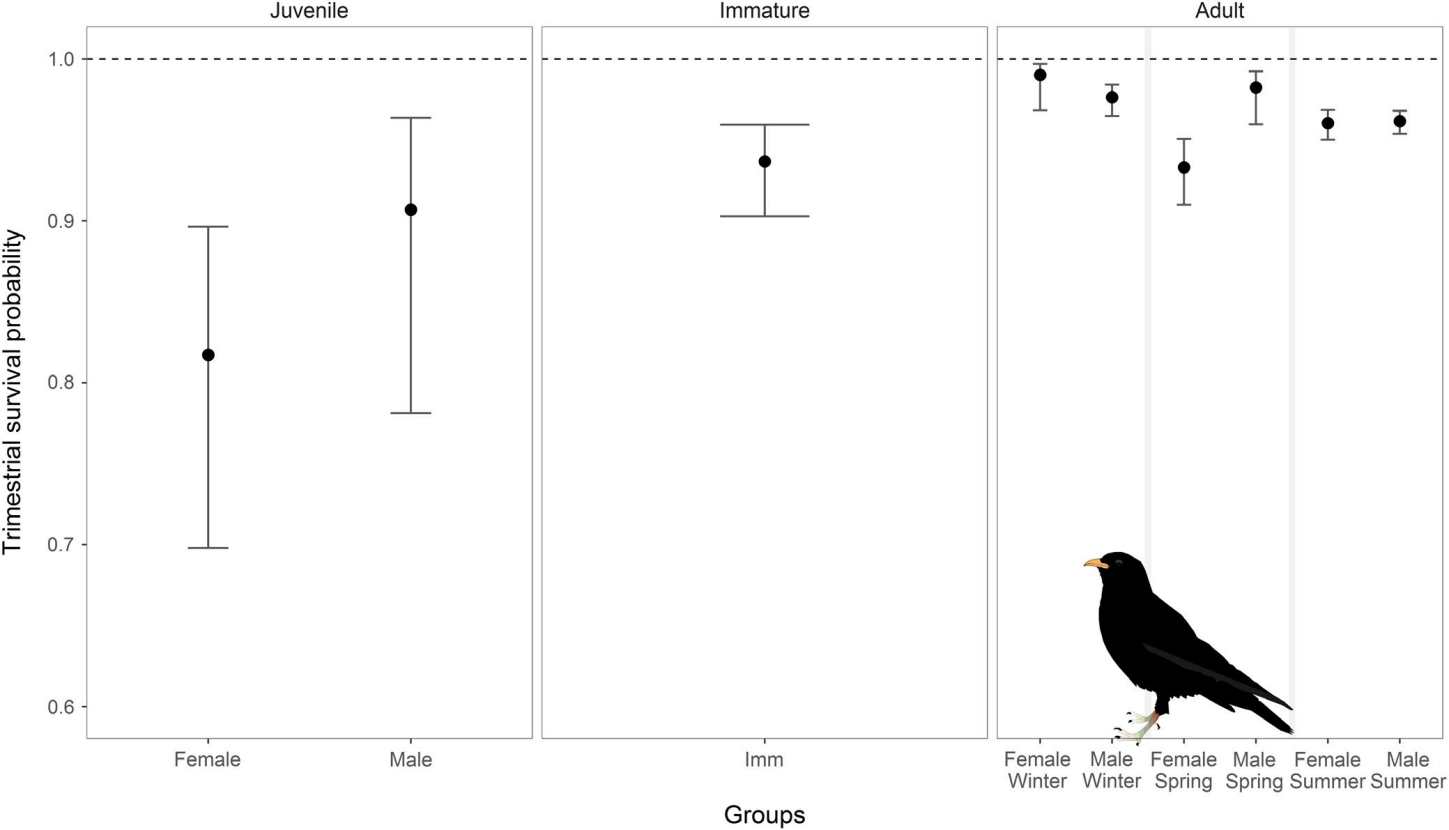
Survival probability of the alpine chough estimated in a multi‐site context in the Mont Blanc region. Survival probability is presented by age class (from left to right), as well as by sex and/or season if considered significant using a delta AIC method. Juvenile survival is based on one summer/winter transition, thus a seasonality effect cannot appear. In immature individuals, no significant seasonal effect was found

The annual fluctuations of alpine chough survival are shown in Figure 1 for each age class. They reveal very large variations in juveniles and immature birds, while the estimates for adults are much more stable across years. Quarterly juvenile survival for summer/autumn and autumn/winter transitions (no data from autumn) was estimated as *φ* = 0.91 [0.77–0.97] for males and *φ* = 0.82 [0.69–0.89] for females. Quarterly immature survival probability was estimated as *φ* = 0.94 [0.90–0.96], for both sex and all seasonal transitions. The probability of an adult male to survive from spring to summer was estimated as *φ* = 0.98 [0.96–0.99], while female survival was estimated as *φ* = 0.93 [0.91–0.95]. Summer to winter survival was very similar for adults of both sexes (*φ* = 0.96 [0.95–0.97]), and lower than winter to spring survival (*φ* = 0.98 [0.97–0.98] for females and *φ* = 0.99 [0.97–0.99] for males, Figure 2). Our models estimated that the alpine chough's first‐year survival probability was 0.58 [0.43–0.71] for females and 0.72 [0.54–0.83] for males, and annual survival probability was 0.85 [0.82–0.87] for adult females and 0.89 [0.86–0.90] for adult males.

### 3.3 Effect of covariates on first‐year survival and adult spring and summer survival

The estimated effects of the covariates in the best models are shown in Table 1 (for the complete table, see Table S2 in Appendix S1). The model accounting for an individual's sex/size group was the best model without covariates and showed high variation in spring survival, even between larger females and smaller males, despite the nearly equal mean tarsus length in these two sex/size groups (mean tarsus length is 44.7 mm for larger females vs. 44.3 mm for smaller males, Figure S3 in Appendix S1). Smaller females also seem to have lower survival probability than larger females during the critical spring/summer transition (−0.02 survival probability, that is, a +25% mortality rate, see Figure S10 in Appendix S1), but the evidence is not strong. This model accounting for an individual's sex/size was very close to the model implemented solely with sex groups in terms of AICc, but with two more parameters, thus the latter was considered the best model and was used for further covariate testing (delta AIC = −0.2, Table 1).

Models accounting for inter‐annual variation in adult survival resulted in higher AICc values than constant models, pointing to low inter‐annual variation in seasonal survival estimates. Immature survival was not correlated to temperature, precipitation or snow anomaly variation occurring during cold seasons. But the cumulative winter and spring temperatures were correlated to variations in adult female survival during the spring/summer transitions, with a negative effect of high temperatures on female spring/summer survival (delta AIC = −3.0, ANODEV *p*‐value = .025 with 28% of variance explained). Adult female survival during spring/summer transitions was also correlated to flock size variation, but the evidence was not strong (delta AIC = −1.5, Table 1, ANODEV test, *p*‐value = .09, explaining 16% of adult female survival variation during the spring/summer transition (Table 1). In years, when the flock size was small (i.e., ≤100 individuals), survival was estimated as about *φ* = 0.95, while it was about *φ* = 0.90 for a mean flock size of ≥140 individuals. No evidence of a correlation between weather covariates and adult survival was found in the summer/autumn transition.

### 3.4 Dispersal

The propensity to disperse from a birth site was much higher in immature females *ω* = 0.87 [0.77–0.92] than in immature males *ω* = 0.49 [0.37–0.62] (Figure S6 in Appendix S1). Immature individuals that dispersed showed a site‐dependent arrival pattern (Figure S7 in Appendix S1). Adult annual departure probability was *ω* = 0.09 [0.08–0.10] for both sexes and all sites. Arrival probability was site‐dependent and sex‐dependent in adults (Figure S7 in Appendix S1).

### 3.5 Resighting probability

Resighting heterogeneity in summer sites was high for sites 1 (Lac Blanc) and 2 (Couvercle). Adults in the high resighting probability group were 6–20 times easier to resight than adults in the low resighting probability group after 10 days of sampling effort (*p* = .90–1.00 vs. *p* = .05–.15, respectively). Immature resighting probability during winter and spring was equal in both sexes and in both seasons *p* = .12 [.09–.17]. During summer, immature recapture probability was much higher than during cold seasons, and differed between summer sites: *p* = .97 [.66–.99] in site 1 (Lac Blanc), *p* = .52 [.25–.79] in site 2 (Couvercle), and *p* = .78 [.48–.93] in site 3 (Albert 1er).

## 4 DISCUSSION

Adult survival is seasonally structured in the alpine chough. Contrary to our expectations, adult survival was higher in winter than in summer. The end of the cold season, which is the spring/summer transition in this area, was a critical survival period for adult females, though not for adult males. Cumulative winter and spring temperatures explained 28% of the variation in female survival in spring, with lower survival during warmer winters and springs.

### 4.1 High survival in the alpine chough

The alpine chough has the highest survival rate in the corvid family (see the multiple species comparision in Ha, Butler, & Robinette Ha, 2010), as well as among alpine passerine species of western Europe (Bastianelli et al., 2017). Alpine chough survival is higher than that of the red‐billed chough (*Pyrrhocorax pyrrhocorax*) (Reid, Bignal, Bignal, McCracken, & Monaghan, 2004), a closely related species sharing most of its traits with the alpine chough, and is higher than that of the western jackdaw (*Coloeus monedula*; Verhulst & Salomons, 2004), a similar‐sized corvid. Both of these species live at lower mean elevations than the alpine chough. Like other alpine species, the alpine chough may have developed life‐history traits that include high investment in self‐maintenance and a “slow” pace of life (Boyce et al., 2006; Hille & Cooper, 2015). This species may also benefit from its high specialization to extreme environments, suffering lower predation/competition pressures than similar species living at lower elevations.

### 4.2 Seasonal survival patterns

The general pattern of survival variation with age is typical for a long‐lived species, with increased survival probability (Figure 2) and decreased survival variation (Figure 1) with the age of individuals (Péron et al., 2016; Sæther & Bakke, 2000). In immature and juvenile individuals, survival varied considerably between years and seasons. This variation in survival may be attributable to lower resistance to environmental harshness in juvenile and immature individuals, or could be interpreted as a bet‐hedging strategy (Gaillard & Yoccoz, 2003). However, we found no correlation between survival and weather conditions during cold seasons in immature individuals, despite the high inter‐annual survival variation. This result, as well as the absence of seasonal patterns in immature survival, might be explained by low statistical power resulting from the addition of high survival variability (Reid et al., 2008) and a low resighting rate of immature birds during cold seasons.

In adult alpine choughs, survival is higher in the harsh winters of the Mont Blanc region than during the summer season. Animals living in polar biomes have developed many physiological and behavioral adaptations to survive harsh seasons, including fat reserves (Douhard, Guillemette, Festa‐Bianchet, & Pelletier, 2018), lower metabolism, or behavioral mechanisms such as optimized foraging, basking, shelter‐building, short‐term elevational displacement to avoid extreme events. (Laiolo et al.., 2001; Marjakangas, Rintamäki, & Hissa, 1984; Martin, 2001; Martin & Wiebe, 2004). For instance, alpine ungulates have high survival in the first months of winter, which may be attributable to stored fat reserves (Gonzalez & Crampe, 2001). The few available seasonal studies on alpine bird species seem to confirm, with low samples, higher survival at the beginning of the cold season than in summer: for example, in the alpine ptarmigan (*Lagopus muta*; Novoa et al., 2011) or the black grouse (*Tetrao tetrix*; Angelstam, 1984). However, unlike grouse, which are “capital breeders”, the active foraging behavior of alpine choughs during winter suggests that this species has adopted an “income” strategy (Stephens et al., 2009). Sociality, high cognitive ability and diet plasticity help the alpine chough obtain resources. These traits allow for complex behaviors such as information sharing, opportunistic foraging on human refuse, and taking advantage of the warmer microclimates of human settlements, as have been shown in other corvids (Seed, Emery, & Clayton, 2009; White, 2005). These behaviors may be critical in allowing the species to remain so high in elevation during the cold alpine winters. As choughs are mainly scavengers in cold seasons, the recent “rewilding” of western European mountains (Navarro & Pereira, 2012) may also be an advantage for their survival in cold seasons, with higher carrion availability in harsh periods.

We found that summer survival is lower than winter survival for both sexes in adult alpine choughs. Cold‐adapted vertebrates are known to be directly or indirectly impacted by warm summer temperatures (Furrer et al., 2016), influencing their foraging strategy (Mason, Brivio, Stephens, Apollonio, & Grignolio, 2017), body condition (Gardner et al., 2016), and survival probability (White et al., 2011). However, we found no evidence of a correlation between summer survival, mean summer temperature, and mean summer precipitation in the alpine chough. The very high diversity of facies in a mountain massif may explain the absence of a direct correlation between weather covariates and survival, as individuals can easily move to find optimal foraging locations and microclimates. Additionally, alpine choughs resort to eating invertebrates or fruits such as bilberries (*Vaccinium myrtillus*) during summer/autumn, which increases their ability to adapt to differing summer conditions. The absence of clear evidence of a direct or carry‐over effect of weather covariates on summer survival suggests that lower survival in summer may be related to a recurrent reproduction and/or molt cost at this period of the year, including higher predation risk during this season (increased predator activity during reproduction, for example, by the beech marten *Martes foina*).

### 4.3 Lower female survival in spring: a matter of social hierarchy and weather?

**Figure 3:**
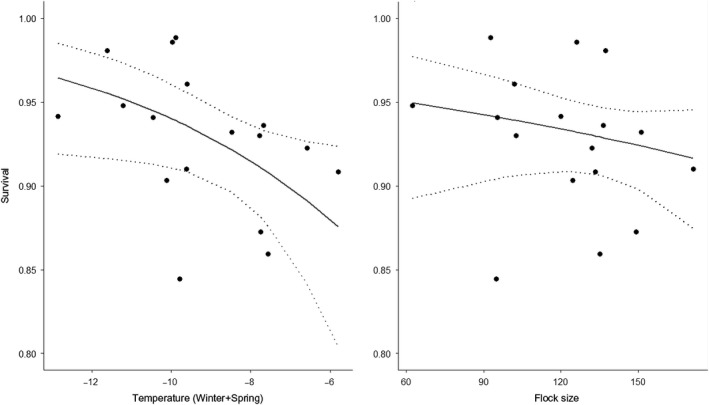
Survival probability of adult female alpine choughs at the end of the cold season as a function of cumulative winter and spring temperatures (left, test value 0.03) and flock size during cold seasons (right, test value 0.09)

While our findings did not detect consequences of harsh alpine winters on alpine chough survival in winter, these may be delayed to spring, the end of the cold season. We found that spring is a critical survival period, but only for females. The lower survival of females in spring might have been caused by emigration outside the study area. Yet we did not detect any dispersal differences between males and females in the three study sites, and the models with sex‐specific adult dispersal showed the same estimates for survival. The model including sex‐size groups indicated that in alpine choughs, females do not have lower survival because they are smaller than males, but mostly because they are females (Figure S10 in Appendix S1). Females in many bird species are known to experience lower survival during the breeding season, which is primarily linked to the higher physiological allocation of energy to reproduction than in males: for example, through egg production (Bize, Godefroy, Patricia, Edoligez, & Christe, 2008; Sæther, Andersen, & Pedersen, 1993; Williams, 1966). Since capture was conducted close to breeding sites, the sample in this study was likely to be mainly composed of individuals involved in reproduction. Additionally, in alpine choughs, males systematically dominate females for food access in clumped food resources (Delestrade, 1993a). This suggests that the social status of females in this species results in their greater sensitivity to environmental constraints, notably during the critical period at the end of winter. The negative effect of flock size on female survival might result from increased intraspecific competition for food access; however, this effect is weak (Figure 3).

Adult female survival in spring was significantly correlated to weather conditions, with lower survival when the cold season was warmer. We found that mortality rates doubled when cumulative winter and spring mean temperatures were between −8° and −6°C compared to years when they were between −12° and −10°C. The negative effect of higher temperatures may seem counterintuitive, but could be explained by an accumulation of physiological constraints. First, warmer winters and springs are characterized by more days of rain than snow. Rain is much more challenging than snow for alpine choughs energy‐wise, as their feathers are not waterproof. Moreover, a warmer winter period may delay the spring phenology of some plants, as has been shown in the Alps and Himalayas (Asse et al., 2018; Yu, Luedeling, & Xu, 2010), and thus delay the development of certain insects. For the alpine region, predictions seem to converge on warmer summers and autumns, and more days with high precipitation in winter. However, uncertainty about the local consequences of global climate change is very high in alpine regions (Rangwala & Miller, 2012), especially regarding precipitation. If winters continue to warm as they have over the 20th century, counterintuitively, conditions could become very harsh for alpine choughs, with many rainy days in winter, and delayed spring phenology for some prey species.

While our results suggest a link between warm temperatures and adult survival in female alpine chough, their survival did not show a clear negative trend (Figure 1). The rate of warming in the region involved a +1.2°C mean temperature increase over the timespan of the study (Beniston et al., 2018; Ceppi, Scherrer, Fischer, & Appenzeller, 2012; Rangwala & Miller, 2012), which is one of the most intense warming rates for mountains in the world, but might not be enough to result in a clear trend in adult female survival given the 6°C variation of mean winter/spring temperatures in the different years of the study (Figure S3 in Appendix S1). High‐elevation regions are expected to experience more intense warming than lowlands over the 21st century (Pepin et al., 2015; Rangwala & Miller, 2012), as was the case in the 20th century. Such rapid warming leads to conservation concerns for alpine species. The results of this study highlight that the consequences of global warming on alpine populations may be very dependent on seasonal changes in local abiotic conditions. Monitoring of both meteorological conditions and demographic parameters in alpine vertebrates may thus be of major importance to detect and better predict consequences of future changes in alpine environments.

### 4.4 Dispersal

The findings show that individuals displayed much more natal dispersal than breeding dispersal (Figure S6 in Appendix S1). We also found that juvenile females disperse more than juvenile males. This pattern may explain the lower survival observed in female juveniles compared to juvenile males. These results fit the general dispersal pattern found in most species of birds, which is traditionally explained by a lower dispersal cost for females, as males defend territories (Clarke, Sæther, & Røskaft, 1997; Greenwood, 1980; Greenwood & Harvey, 1982).

## 5 CONCLUSION

This study used a robust two‐step approach to assess the influence of environmental constraints on the demography of a species inhabiting a seasonal environment. This first full season‐cycle analysis of survival in a sedentary bird inhabiting an alpine biome revealed very high winter survival, lower summer survival, and contrasting spring survival, depending on the sex of individuals. It would be very useful to test if this seasonal pattern is shared with other alpine and arctic species. The impact of weather conditions on the survival of socially dominated individuals, in this case females, at the end of the cold season is likely to be a general phenomenon, but its importance should be assessed in other species and ecosystems as well.

## CONFLICT OF INTEREST

None declared.

## AUTHORS CONTRIBUTION

AD, AB, NGY, and JC conceived the main hypothesis; AD collected the data; AB designed the analysis methodology; JC analyzed the data; JC led the writing of the manuscript, and all authors contributed critically to the drafts and gave final approval for publication.

## Data Availability

Dataset on individuals' capture/recapture history by season is on the Dryad repository, https://doi.org/10.5061/dryad.k173v65.
