# Supplementary figures and images for: Warm temperatures during cold season can negatively affect adult survival in an alpine bird
Source: Ecol Evol. 2019 Oct 25;9(22):12531–43. doi: 10.1002/ece3.5715 (PMC6875669; doi:10.1002/ece3.5715)

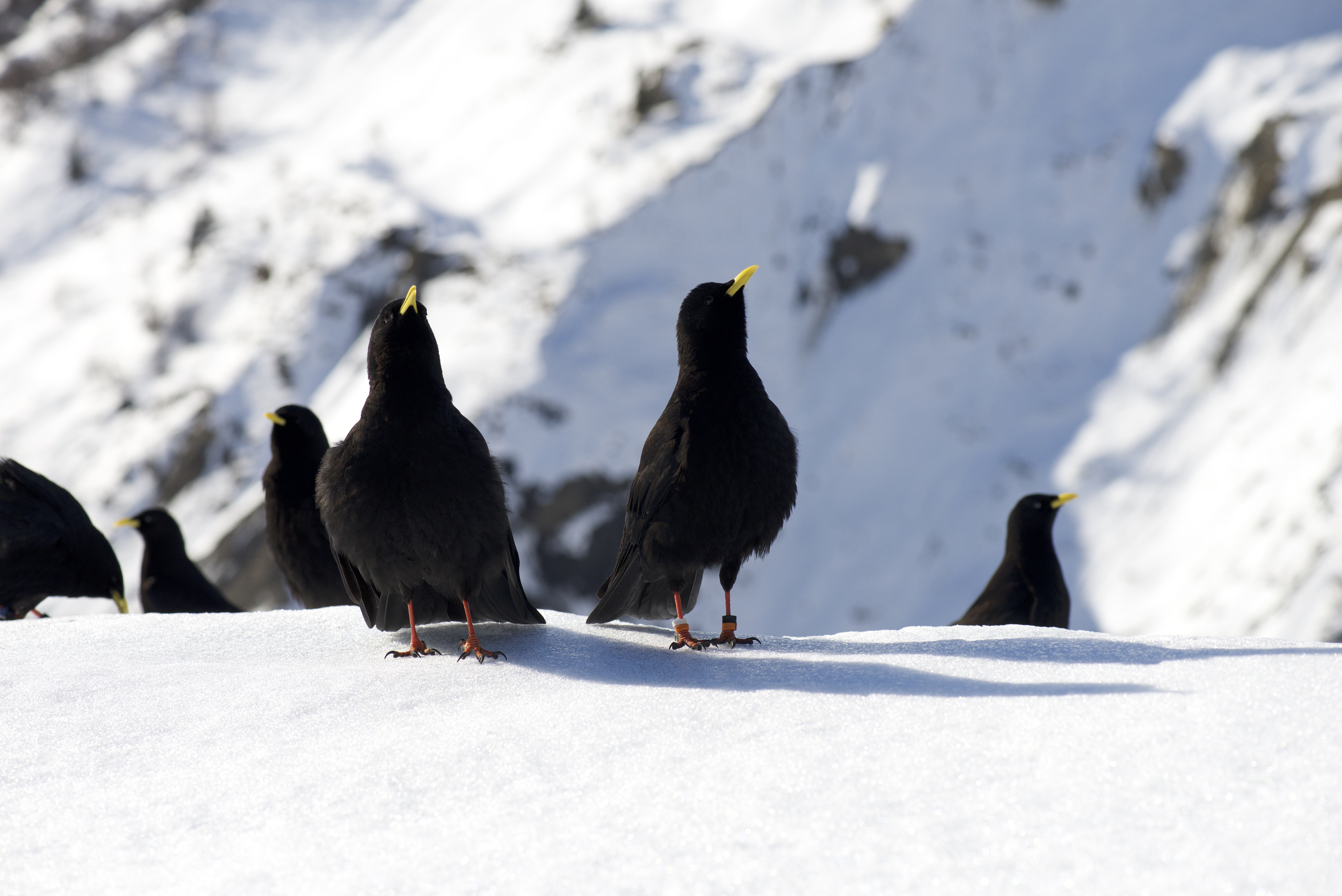

Supplement: Supplementary file 1 [file ECE3-9-12531-s001.png]
