# Supplementary material for: Warm temperatures during cold season can negatively affect adult survival in an alpine bird
Source: Ecol Evol. 2019 Oct 25;9(22):12531–43. doi: 10.1002/ece3.5715 (PMC6875669; doi:10.1002/ece3.5715)
Supplement: Supplementary file 2 [file ECE3-9-12531-s002.docx]

**Supplementary Material Appendix S1** *:*

Table S1: Results of the goodness of fit tests on simple datasets (only adults, only one season, multi-site).

STA: Test’ statistic; PVAL: P-value associated with the test; DF: degrees of freedom


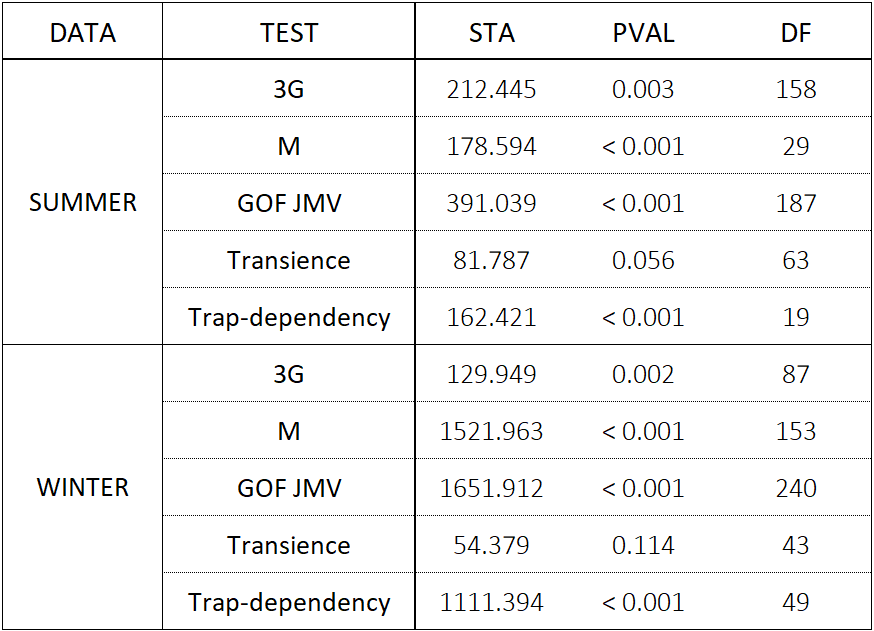


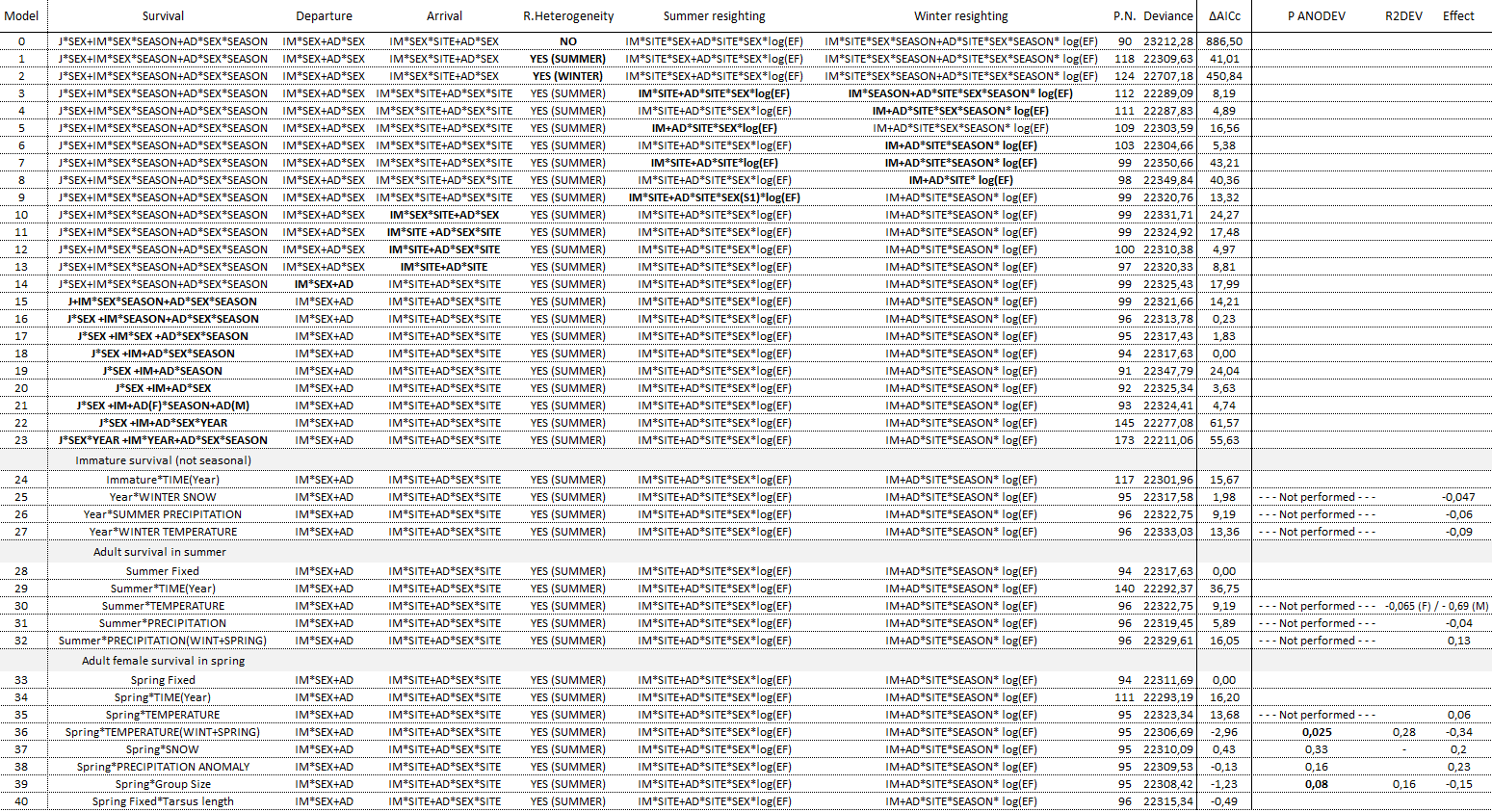
Table S2: Model selection matrix showing the models that best describe alpine chough survival. Formulas include stages and their respective effects, as an example when survival is noticed *AD*SEASON*SEX+IMM*SEX* means that season and sex effects are in interaction on adults’ survival but only a sex effect is implemented for immature survival that is thus constant over seasons*.* **Bold series of parameters are those which were simplified in our procedure.** RH= Resighting heterogeneity; P.N. = Estimated parameters number; J = juveniles; IM = immature individuals; AD = adults; (F) = females; (M) = males; EF = effort; K = number of estimated parameters; Dev: model residual deviance; AIC = Akaïke Information Criterion; S1= site 1; SEASON = differences between seasons; + = additive effects; * = effects in interaction.


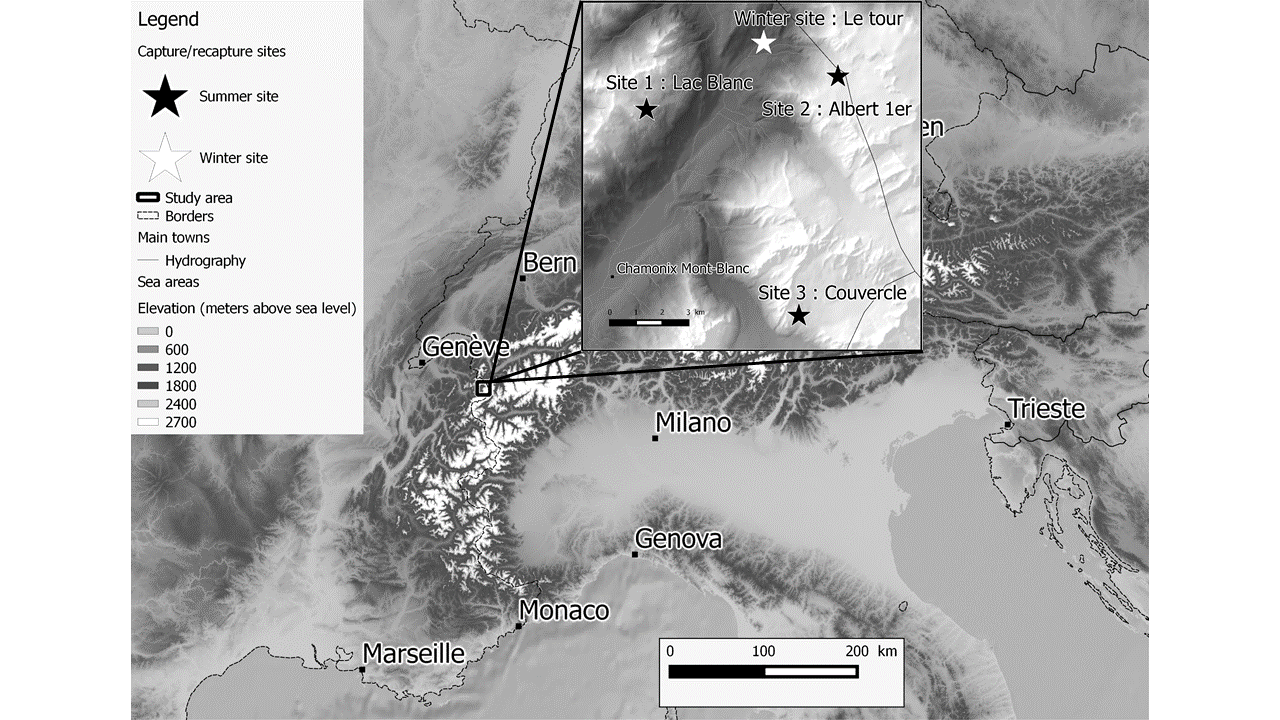

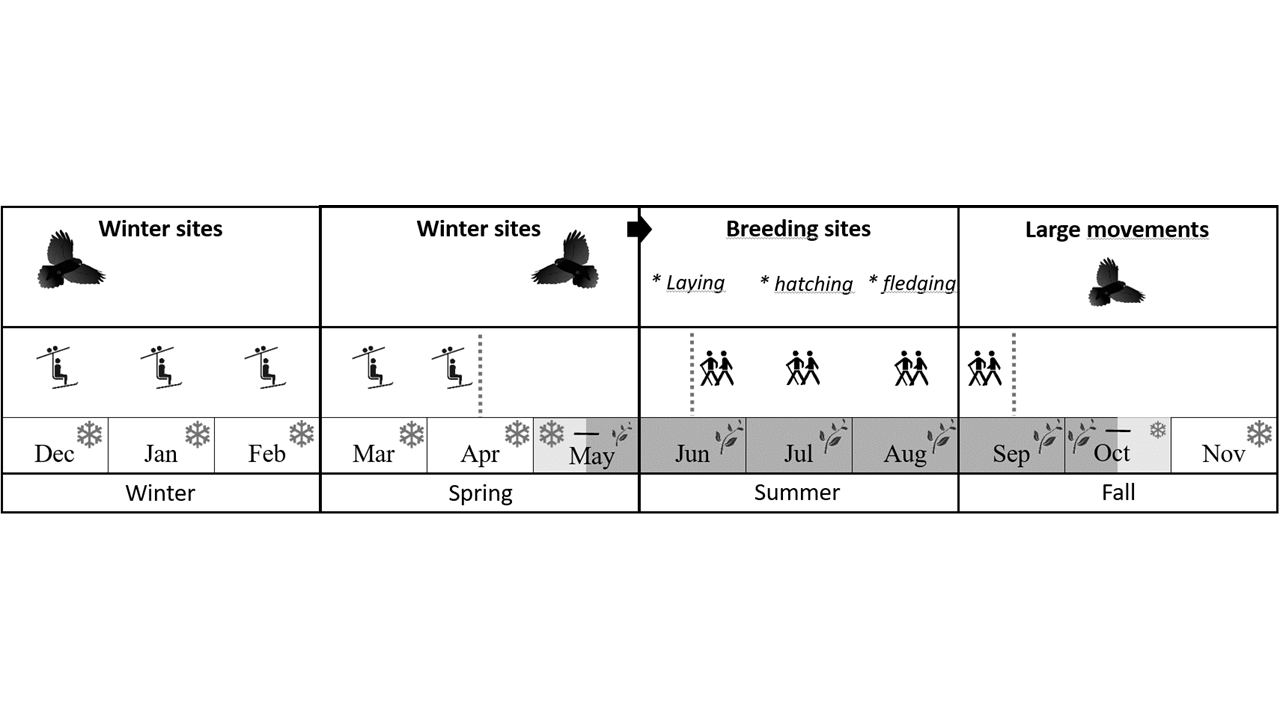


Figure S1: (Up left) Location of the study site in the Western Alps. From 1988 to 2014, 1095 alpine choughs were captured, marked and resighted at three summer sites and one wintering site. (Bottom) The phenology of the Mont Blanc study area by season: seasonal lifecycle of alpine choughs (top row), main human activities (middle row), including skiing in winter/spring and hiking in summer, snow or plant cover (bottom row), with shading indicating the amount of vegetation present.


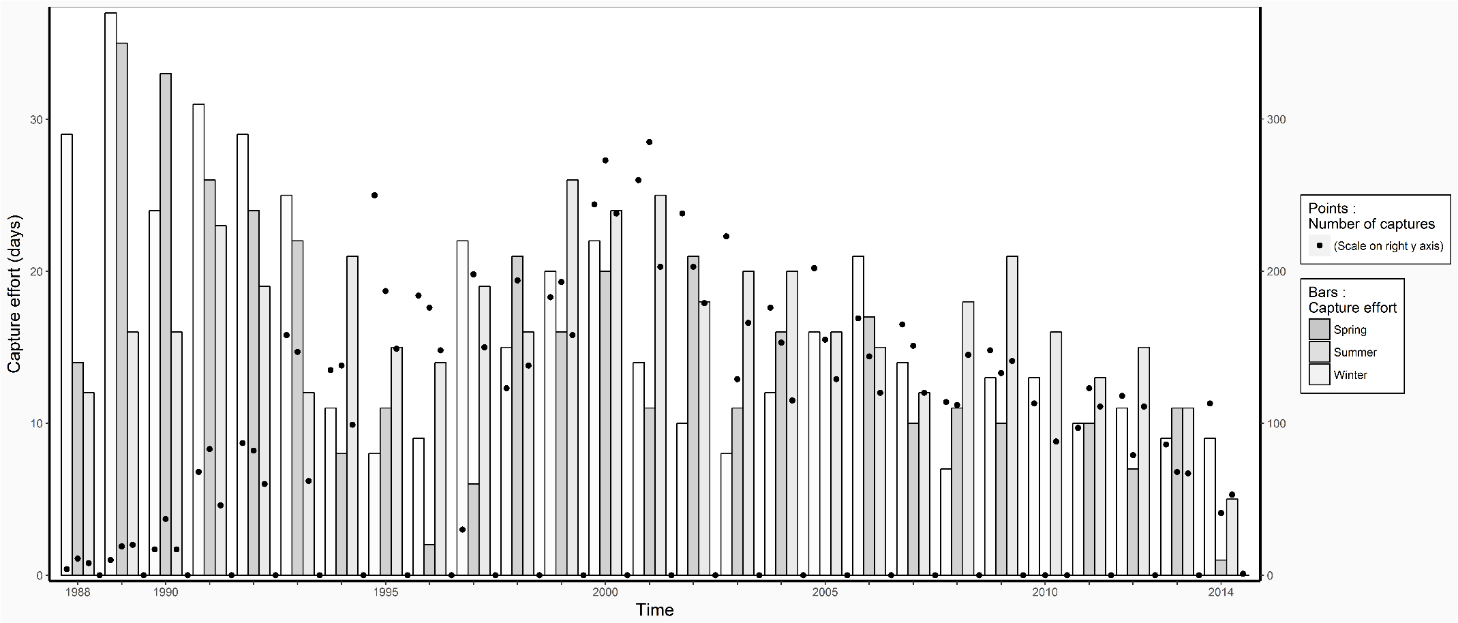


Figure S2: Capture effort (number of days) and number of alpine chough individuals captured in each season per year over the study period.


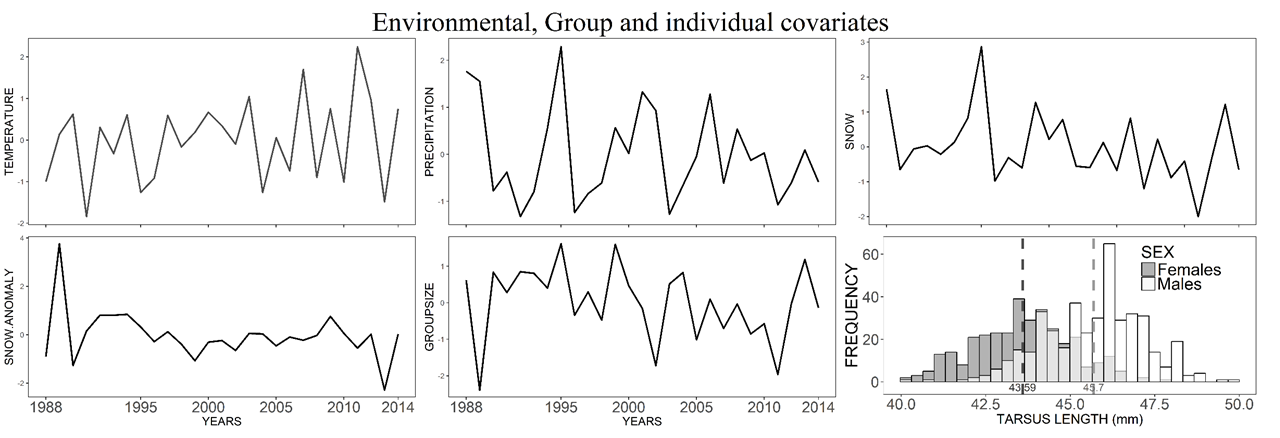


Figure S3: Temporal variation of environmental covariates in the Mont Blanc region during the study period, and the frequency of tarsus length in alpine chough individuals by sex.


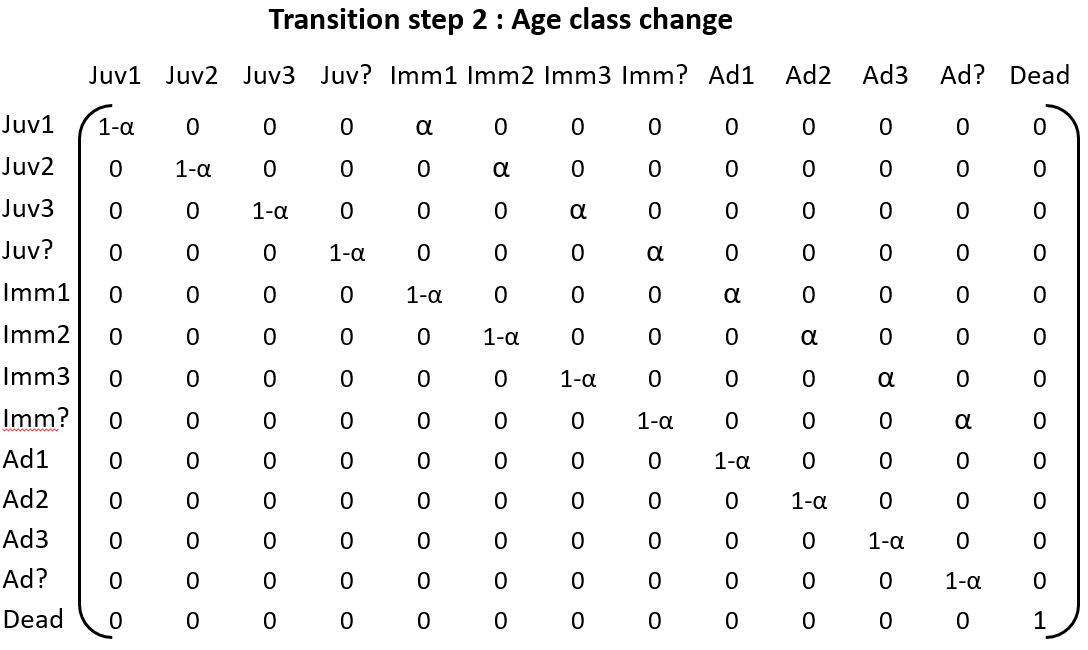

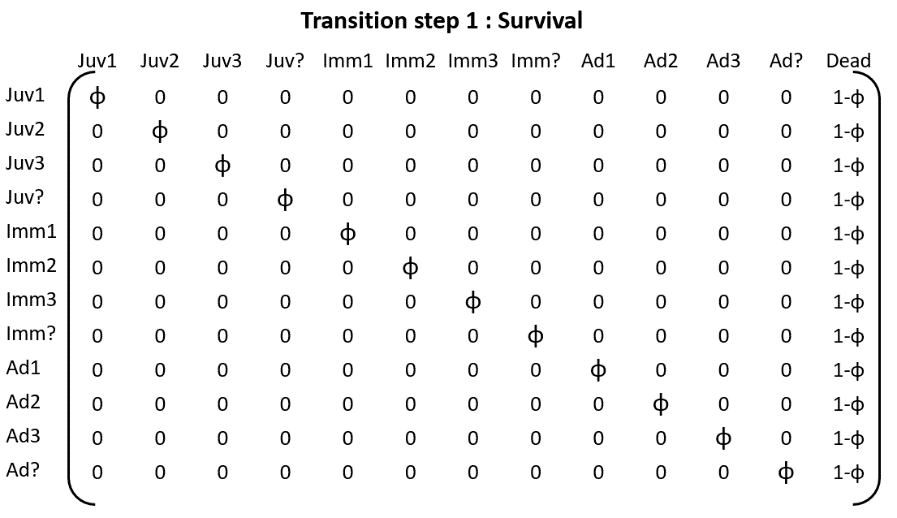

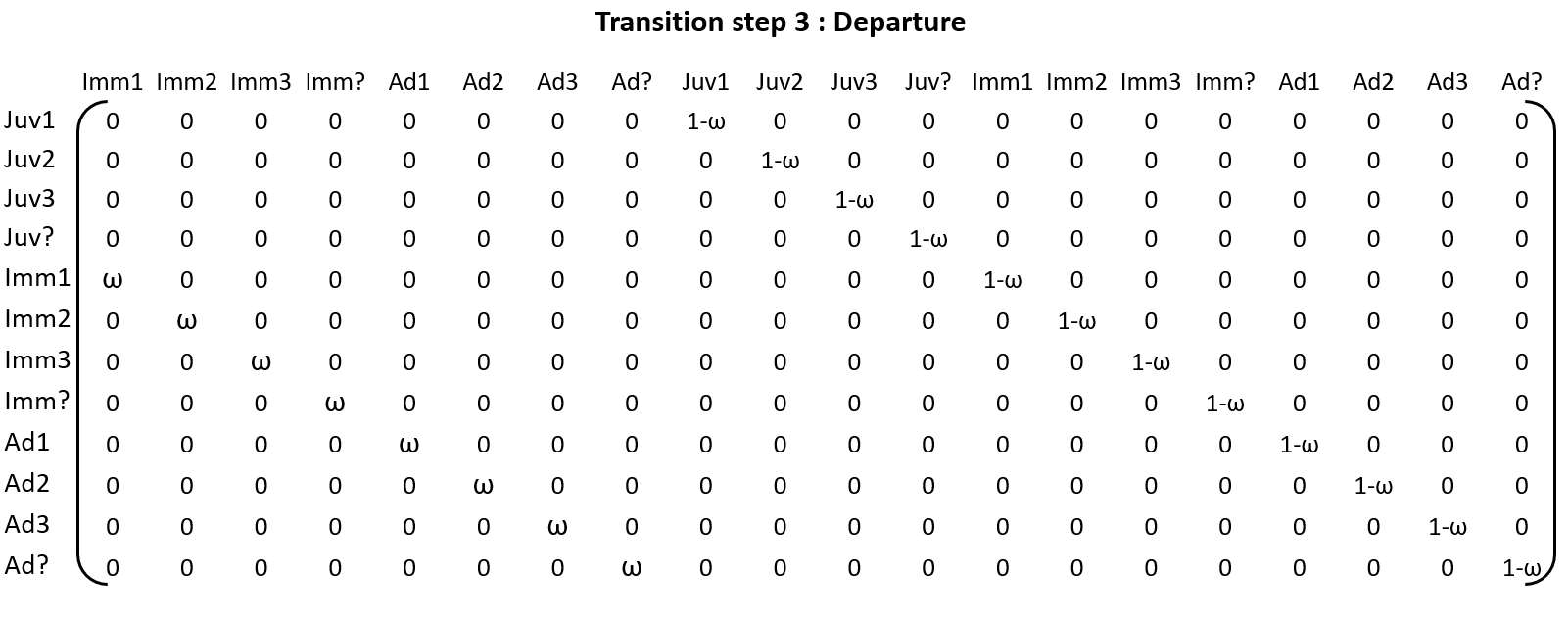

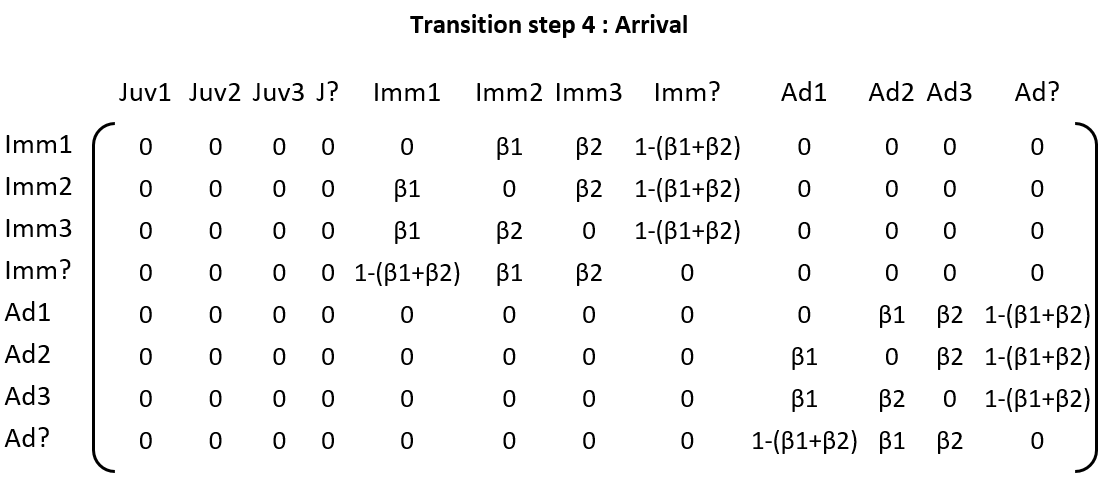

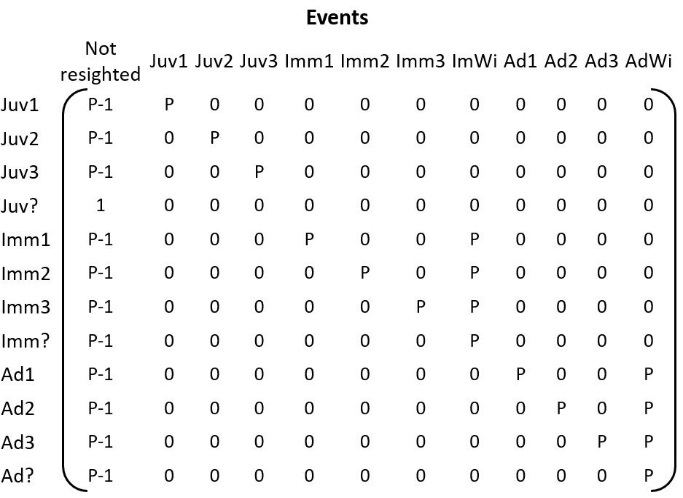

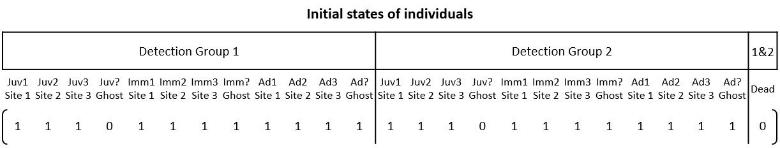


Figure S4: Matrices of multievent capture–recapture models fitted for the data (initial states, transitions and events). ‘Juv’, ‘Ad’ and ‘Imm’ refer to age classes (juveniles, adults and immature individuals), and numbers refer to the summer site occupied (‘?’ refers to a ‘ghost’ or unknown summer site). States assigned with ‘0’ in the initial state (i.e. state at first capture) exist, but never occurred in the dataset (dead individuals were not ringed, nor were juveniles in unknown sites). Parameters of interest are φ: survival probability, α: age class transition probability (forced to 0 or 1 depending on the season), ω: departure probability, β: arrival probability and P resighting probability. In the events matrix, rows represent an individual’s states and columns the observations from resighting efforts. A ‘0’ was assigned for impossible observations. Age classes were always identified, but during winter, the summer site could not be identified, resulting in state uncertainty.


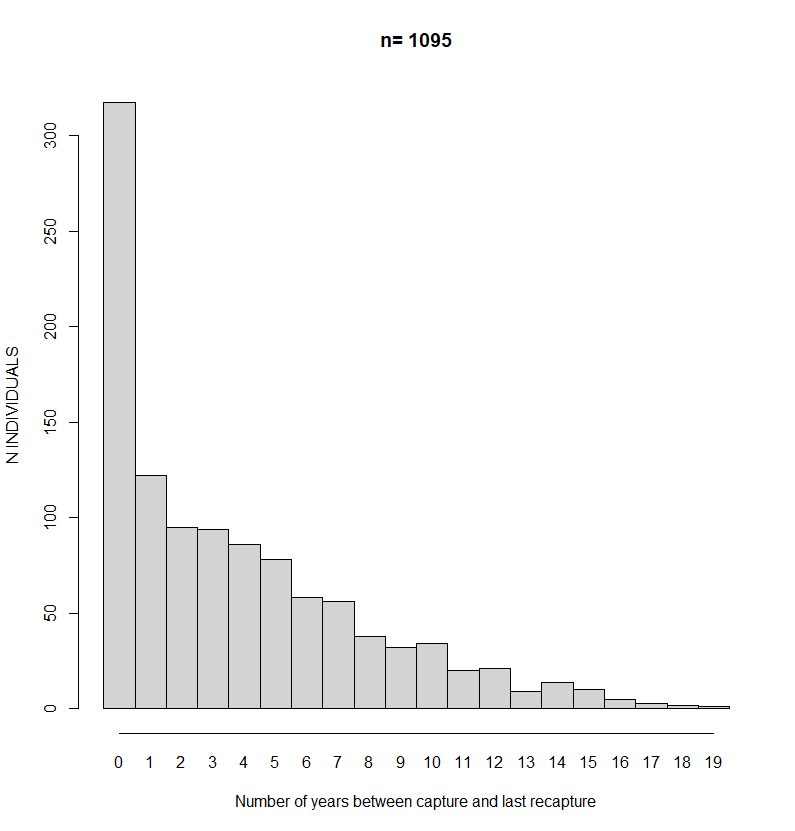


Figure S5: Number of years between capture and last recapture for alpine chough individuals in the study.


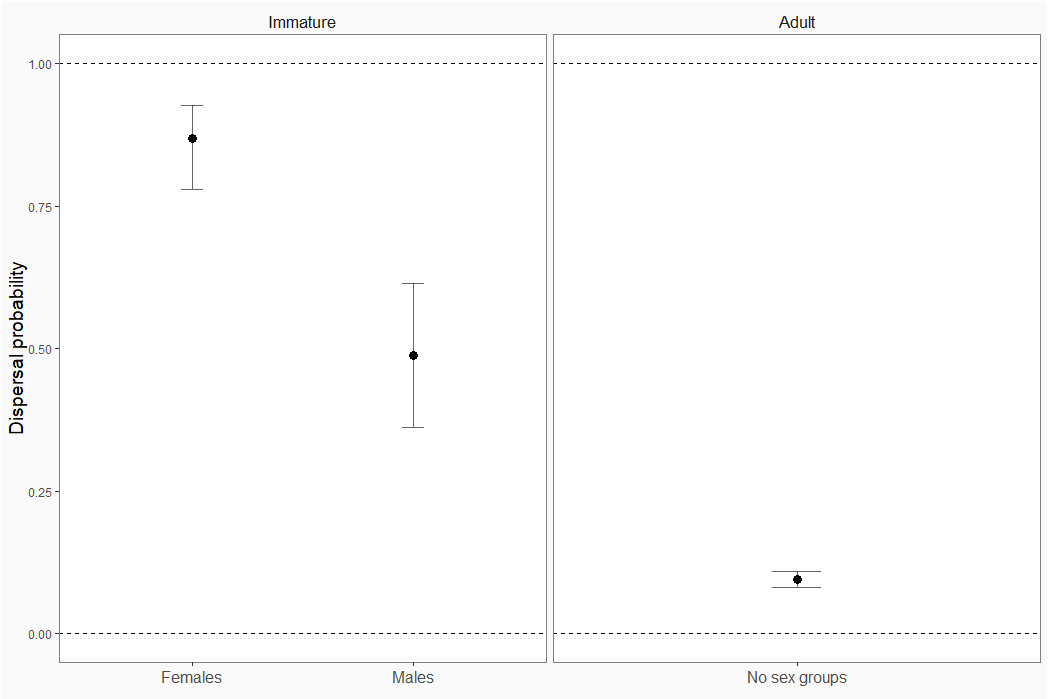


Figure S6: Dispersal probability of the alpine chough by age class: first-year dispersal (on the left) is sex dependent, while adult dispersal (on the right) is not.


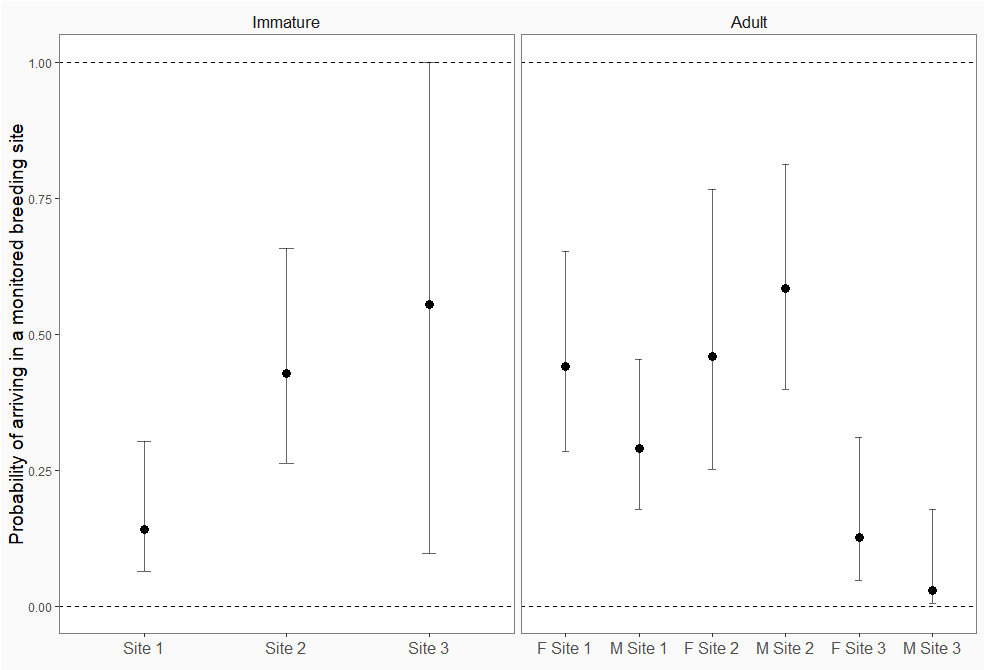


Figure S7: Arrival probability in one of the three monitored sites by age class and (for adults) sex, with the 95% confidence intervals.


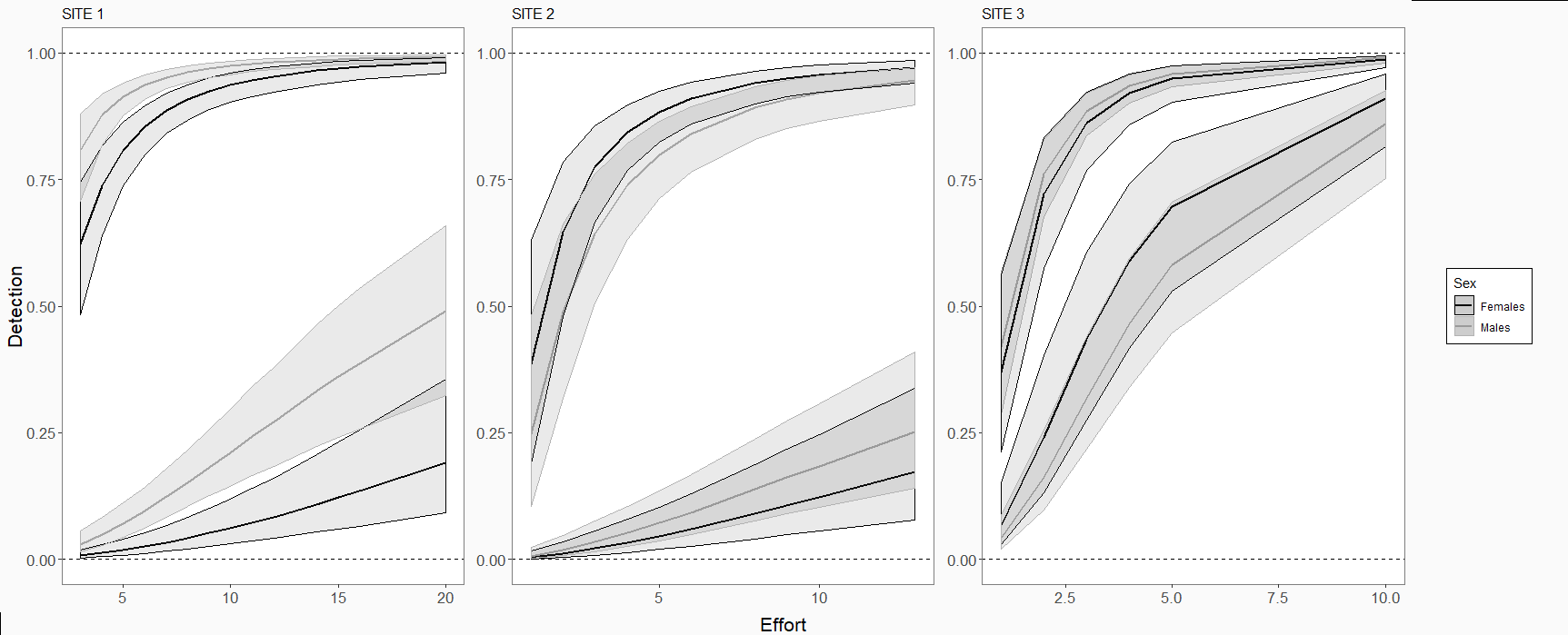


Figure S8: Resighting probability of adult alpine choughs in summer sites depending on capture effort, with 95% confidence intervals. For each sex and site, hidden heterogeneity groups classified individuals depending on their detection probability in summer. In sites 1 and 2, almost half of individuals were classified in both heterogeneity groups. In site 3, most individuals are in the most detected group.


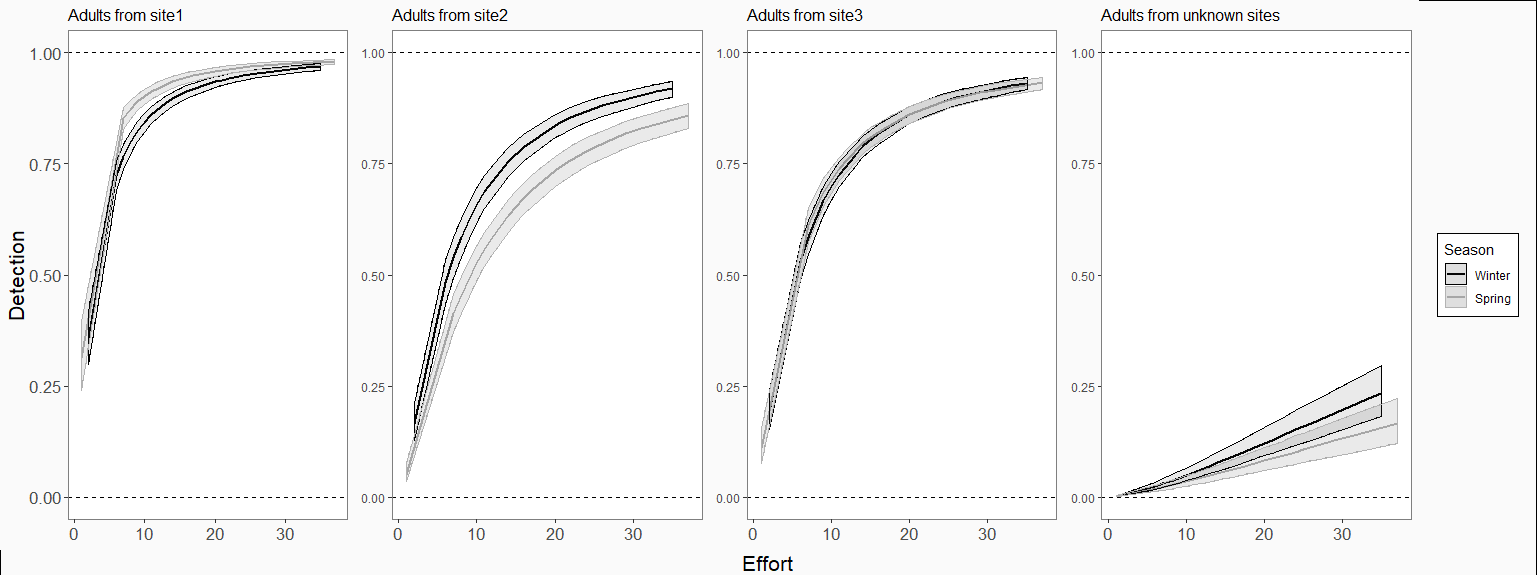


Figure S9: Resighting probability of adult alpine choughs in the cold seasons depending on the summer site occupied and season, with 95% confidence intervals.


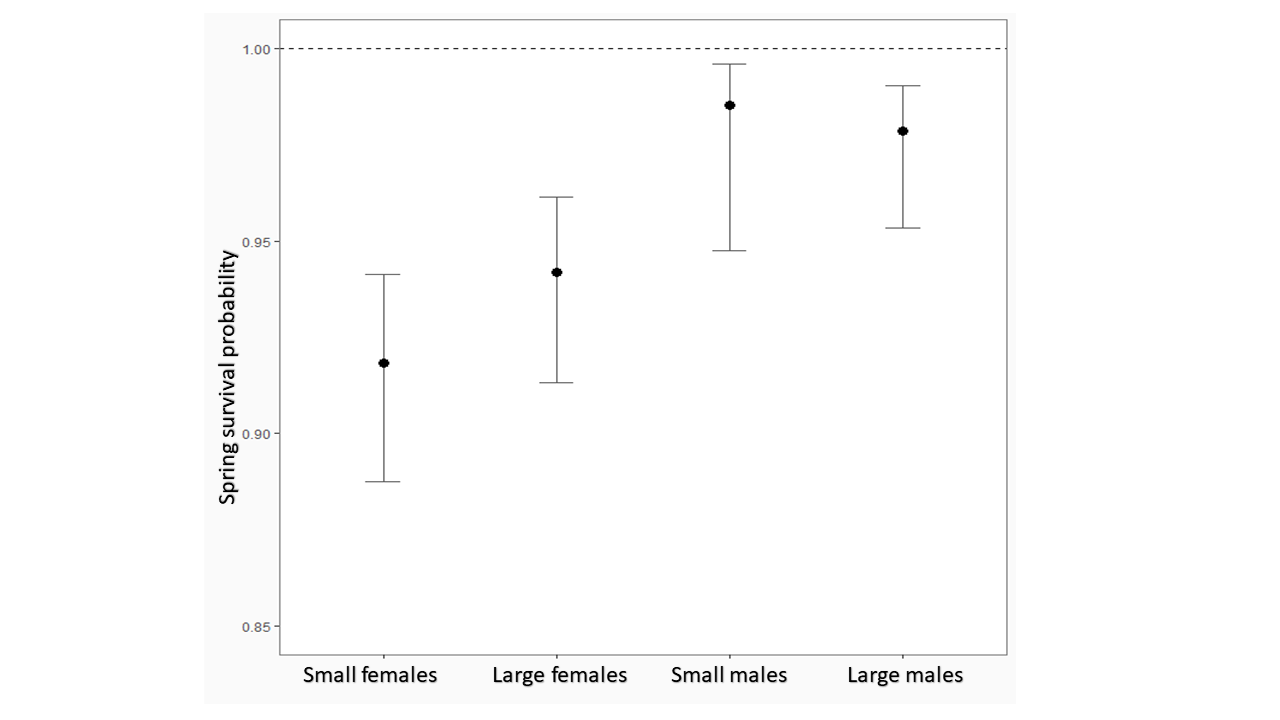


Figure S10: Spring survival probability with 95% confidence intervals for alpine choughs by size and sex. On average, large females are a similar size to small males.
